# Moderate to Vigorous Physical Activity as a Cause of Dietary Restriction in Eating Disorders

**DOI:** 10.1002/eat.24494

**Published:** 2025-06-27

**Authors:** K. Jean Forney, Angela R. Hillman, Lindsay P. Bodell

**Affiliations:** ^1^ Department of Psychology Ohio University Athens Ohio USA; ^2^ Department of Exercise Physiology Ohio University Athens Ohio USA; ^3^ Department of Psychology University of Western Ontario London Ontario Canada

**Keywords:** energy intake, exercise, feeding and eating disorders, ghrelin, glucagon‐like peptide 1, peptide YY, physical activity, reward

## Abstract

**Objective:**

The mechanisms that facilitate prolonged dietary restriction in eating disorders, particularly in the absence of binge eating, remain poorly understood. The activity‐based anorexia model and basic science in exercise physiology suggest that moderate to vigorous physical activity leads to reduced energy intake relative to metabolic needs. This reduction in energy intake is even greater when individuals exercise in the fasted, compared to fed, state.

**Method:**

We propose a model in which moderate to vigorous physical activity facilitates increased dietary restriction within eating disorders. We propose that moderate to vigorous physical activity, regardless of motivation, reduces ghrelin, increases glucagon‐like peptide 1 and peptide YY, and reduces food reward. These mechanisms, in turn, contribute to reduced relative energy intake.

**Results:**

We review relevant rodent and human literatures to evaluate the model and identify observational and experimental research designs to test these hypotheses.

**Conclusion:**

Understanding how and when moderate to vigorous physical activity contributes to dietary restriction has important implications for tailoring eating disorder treatment.

**Summary**

- Moderate to vigorous physical activity acutely reduces relative energy food intake in healthy populations.
- The effect of physical activity on relative energy intake is stronger when individuals engage in fasted exercise.
- The current paper proposes a mechanistic model for how moderate to vigorous physical activity may facilitate restrictive eating.

## 1 Introduction

**FIGURE 1:**
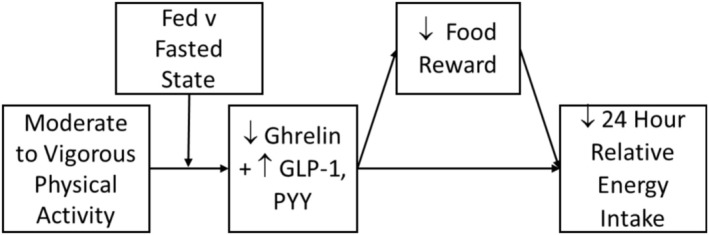
Proposed model of mechanisms underlying the link between exercise and dietary restriction.

The mechanisms that facilitate prolonged dietary restriction remain poorly understood, particularly in the absence of binge eating, as in anorexia nervosa, restricting type, atypical anorexia nervosa, and purging disorder. Patients with eating disorders appear to override a potent orexigenic signal, elevated ghrelin (Cummings and Overduin 2007). Elevated ghrelin levels are present across eating disorder presentations and weight statuses, whether or not binge eating is present (Breithaupt et al. 2020; Keel et al. 2018; Prince et al. 2009). How do patients persistently override these acute signals? We propose that the physiological consequences of moderate to vigorous physical activity (MVPA) maintain dietary restriction over time by acutely reducing appetitive signals, bolstering satiety and satiation signals, and reducing food reward value. These dynamic changes within individuals are proposed to facilitate dietary restriction, even when an individual's average ghrelin level or reward sensitivity is elevated compared to healthy populations. Further, we propose that the impact of exercise is stronger when individuals exercise in the fasted, compared to the fed, state. We set forth a model of the physiological consequences of MVPA in eating disorders and propose tests of this model (see Figure 1).

## 2 Moderate to Vigorous Physical Activity Reduces Relative Energy Intake, Especially in the Fasted State

MVPA, including cardiovascular exercise like brisk walking or running (colloquially known as “cardio”), is a known cause of reduced energy intake, with effects observed up to 24 h (Bachman et al. 2016; Deighton et al. 2012). A meta‐analysis of 25 experimental trials using objectively measured physical activity and energy intake demonstrated that MVPA reduces relative energy intake (*d* = −1.35, Schubert et al. 2013). MVPA does not cause a reduction in the absolute amount of energy intake (e.g., total kcal consumed; Schubert et al. 2013). Instead, MVPA reduces relative energy intake when energy expenditure is accounted for (e.g., [total kcal consumed] – [energy expenditure during exercise]). Based on our clinical experience, it seems likely that patients often fail to increase intake sufficiently to compensate for physical activity, making MVPA a clandestine facilitator of dietary restriction. Indeed, healthy populations compensate by eating more after energy deficits caused by food restriction, but not for energy deficits caused by MVPA (King et al. 2011). Although the majority of this research has been conducted in men, meta‐analysis suggests that the effect does not differ by sex (Schubert et al. 2013).

The effects of MVPA are amplified when individuals are in the fasted, compared to the fed, state with a large effect size (*d* = −1.45; Bachman et al. 2016; Frampton et al. 2022). Our clinical experience suggests that some patients delay eating for hours and often exercise before eating, consistent with high rates of fasting in eating disorder populations (Stiles‐Shields et al. 2012; Wilkinson et al. 2024). We propose that MVPA in the fasted state is more potent in facilitating dietary restriction than MVPA in the fed state.

## 3 Proposed Mechanisms

We propose that MVPA reduces food intake by reducing ghrelin levels, bolstering peripheral satiety and satiation signals (e.g., glucagon‐like peptide 1 (GLP‐1) and peptide YY (PYY)), and reducing food reward. Fasting ghrelin is elevated across eating disorders (Breithaupt et al. 2020; Keel et al. 2018; Prince et al. 2009), an adaptive response to weight suppression (Keel et al. 2017; Lowe et al. 2011). Because ghrelin is an appetitive signal (Cummings and Overduin 2007), elevated fasting ghrelin levels should correspond to increased food intake. We propose that increases in lactate and free fatty acid (FFA) concentrations resulting from MVPA (Gribble and Reimann 2019; Hazell et al. 2016; Islam et al. 2017; Vanderheyden et al. 2020) reduce ghrelin levels, facilitating continued dietary restriction. Meta‐analyses of healthy populations support that MVPA decreases ghrelin (Anderson et al. 2021; Douglas et al. 2016; Hu et al. 2023; Schubert et al. 2014), with a small trial documenting effects in women with bulimia nervosa (Smitka et al. 2019). Ghrelin suppression is sustained up to 24 h after exercise (Deru et al. 2023). Effects are stronger with more intense cardiovascular activity than resistance training (e.g., weightlifting; Anderson et al. 2021; Douglas et al. 2016; Hu et al. 2023). This effect is present across the weight spectrum and may be stronger for those with a higher BMI (Douglas et al. 2016). We hypothesize that relative decreases in ghrelin facilitate dietary restriction over time, such that this dynamic process explains food restriction in the presence of elevated fasting ghrelin levels observed in eating disorders.

In addition to reducing appetitive signals, MVPA may bolster satiety and satiation signals such as GLP‐1 and PYY (Cummings and Overduin 2007) via increased lactate and FFAs (Gribble and Reimann 2019; Hazell et al. 2016; Vanderheyden et al. 2020). Overall, data are mixed as to whether and how fasting GLP‐1 levels are disrupted across eating disorder diagnoses (Bodell and Forney 2020; Geliebter et al. 2008), with some evidence suggesting lower GLP‐1 levels associated with binge eating (Dossat et al. 2015). PYY is elevated in anorexia nervosa and atypical anorexia nervosa (Bodell and Forney 2020; Muhammed et al. 2025), whereas PYY does not appear to be altered in eating disorders with binge eating (Bodell and Forney 2020; Geliebter et al. 2008). Meta‐analyses suggest that acute exercise increases GLP‐1 and PYY (Schubert et al. 2014), although this effect may not generalize to individuals with overweight/obesity (Douglas et al. 2016). A small trial of women with bulimia nervosa demonstrated an increase in PYY after MVPA (Smitka et al. 2019). Increased GLP‐1 and PYY might lead to increased satiety and decreased food intake (Cummings and Overduin 2007). While cholecystokinin also represents a meal‐related signal, we are unaware of studies assessing the acute effects of MVPA compared to rest on cholecystokinin levels.

Gut peptides may have a direct and/or an indirect impact on appetite via changes in food reward value. In rodents, high intensity exercise causes decreased lever pressing for high fat food (“wanting;” Kirkpatrick et al. 2022). Similarly, high fat food intake is reduced in the activity‐based anorexia model (Brown et al. 2008). Animals in the activity‐based anorexia model also demonstrate decreased preference for sweet taste (Hurley et al. 2022), with one study documenting aversion to sweet stimuli with the introduction of wheel running (Milton et al. 2018).

In the human literature, MVPA acutely reduces implicit “wanting” for high fat foods (Riou et al. 2019). Neural indicators of food reward in normal weight and overweight samples without eating disorders show similar patterns in response to MVPA (Crabtree et al. 2014; Evero et al. 2012; Hanlon et al. 2012). MVPA, compared to rest, is associated with decreased BOLD response in the insula, orbitofrontal cortex, and posterior cingulate cortex (Crabtree et al. 2014; Evero et al. 2012) as well as a decreased late positive potential (Hanlon et al. 2012) in response to food images. Problematically, implicit assessment of food reward value does not necessarily translate to the consumption of rewarding foods. Because reward processing may be altered in people with eating disorders (Bodell and Racine 2023; Leenaerts et al. 2022), it is vital to test whether MVPA might acutely reduce food reward value using assessments of consummatory behavior in eating disorder populations (Bodell and Keel 2015; Keel et al. 2022).

We propose that the link between MVPA and reduced food reward value is mediated by changes to peripheral gut peptides, particularly ghrelin. Peripheral ghrelin administration increases preferences for palatable (rewarding) foods in rodents (Disse et al. 2010; Shimbara et al. 2004), even when satiated (Skibicka et al. 2012). Ghrelin receptor antagonists also reduce bar pressing for sucrose (Skibicka et al. 2012). Among healthy adult men, peripheral ghrelin administration increases neural response to food images in reward regions (Malik et al. 2008). A recent meta‐analysis of fMRI data in humans provides evidence that endogenous ghrelin levels are positively correlated with activation in reward regions when viewing rewarding food images (Schulz et al. 2023).

Data are less consistent linking GLP‐1 and PYY to food reward (Woodward et al. 2022). In rodents, peripherally administered long‐acting GLP‐1 receptor agonists reduce lever pressing and food‐related conditioned place preference (Dickson et al. 2012). While GLP‐1 receptor agonists reduce neuronal activation in response to images of high calorie and/or palatable foods in humans (Badulescu et al. 2024), endogenous GLP‐1 is not consistently associated with suppressed reward responses in humans (Schulz et al. 2023). These discrepancies might reflect that GLP‐1 receptor agonists are longer acting than endogenous GLP‐1. Data are considerably more sparse in considering PYY and its link to food reward (Woodward et al. 2022); it may be that PYY does not play a role in any hedonic processes proposed to link MVPA and reduced energy intake.

## 4 Scope, Limitations, and Alternatives of the Proposed Model

The proposed model is limited to exercise as a *cause* of dietary restriction. Phenotypically, maladaptive and vigorous exercise is a common feature of eating disorders that is often conceptualized as a weight/shape or mood regulation strategy (Bratland‐Sanda et al. 2010, 2011; Gorrell et al. 2021). However, case‐control studies and large observational studies indicate that increased physical activity *precedes* the development of eating pathology (Davis et al. 2005; Davis et al. 2018). In the activity‐based anorexia animal model, weight loss is only observed when the animal has reduced access to food *and* access to a running wheel; restricting access to food alone is insufficient (Spadini et al. 2021). Because exercise might be both a cause and a consequence of eating disorder behaviors, cross‐sectional, correlational studies are not strong tests of the proposed model.

The physiological consequences of exercise are only one of the many factors that play a causal role in dietary restriction (e.g., affect, hormones, and cognitions). Although physical activity may have multiple motivators (e.g., compensation for binge eating, affect regulation, reduction of cardiovascular disease risk, enjoyment, or job requirements; Barker et al. 2024; Mond and Gorrell 2021), we propose that both adaptive *and* maladaptive MVPA contribute to dietary restriction. The model is specific to dietary restriction (i.e., eating less than is needed for daily energy needs) and not dietary restraint (i.e., attempts to limit food intake) (e.g., Stice et al. 2010). By elucidating a circumscribed, causal model that is testable, we hope that findings are more easily translated to clinical practice.

Clinical intuition suggests that MVPA leads to compensatory binge eating. However, in the activity‐based anorexia model, physical activity leads to reduced consumption of sucrose and high fat foods (Brown et al. 2008; Milton et al. 2018), the macronutrient content profiles most relevant to binge eating. Exercise interventions reduce binge eating (Beaulieu et al. 2020; Blanchet et al. 2018; Raisi et al. 2023), above and beyond the effects of cognitive behavioral therapy (Pendleton et al. 2002). Although prior treatment studies cannot elucidate the mediators of this effect (e.g., affect, ghrelin), our proposed mechanisms are candidates. Because our proposed model isolates the physiological consequences of exercise, regardless of psychological context, studies that separate maladaptive and non‐maladaptive exercise (e.g., Wons et al. 2023) would be inappropriate to test this model.

The current model is proposed to apply to all eating disorders. However, trait‐based moderators of these effects are likely. For example, the effects of MVPA on ghrelin may be stronger in individuals with greater adiposity (Anderson et al. 2021; Douglas et al. 2016) or a *U*‐shaped curve may exist between adiposity and MVPA‐induced appetite suppression. Those who are more malnourished may utilize more lactate during physical activity (Kreisberg et al. 1970), leading to greater ghrelin suppression.

Individuals with anorexia nervosa tend to have reduced reward responding to food images (Monteleone et al. 2018), despite elevated ghrelin levels. Because reward responses to unanticipated sweet taste is intact, this general reduction in reward responding may reflect learned fear responses or other cognitive processes (Frank 2015; Monteleone et al. 2018). Pending successful testing of the model, next steps might include examining the interaction of cognition with the physiological consequences of MVPA or the effects of recurrent MVPA on receptor regulation (Pardo et al. 2010).

## 5 Discussion, Future Research and Potential Clinical Implications

We have reviewed basic science from rodent and human models that suggests that MVPA decreases relative energy intake through the suppression of ghrelin and augmentation of GLP‐1 and PYY (see Figure 1). We proposed that these gut peptide changes, particularly changes to ghrelin, also reduce food reward value. We hypothesize that these effects are greater when individuals exercise in the fasted, compared to fed, state.

Both naturalistic and tightly controlled laboratory studies are needed to understand how MVPA might lead to dietary restriction within eating disorders. Intensive observational data that objectively measure physical activity, such as the pairing of ecological momentary assessment and actigraphy data, are vital to testing the hypotheses that MVPA does not increase (or may attenuate) binge eating and that this effect is stronger in fasted, compared to fed, exercise. These observational studies will allow for ecologically valid inferences. Randomized crossover designs that tightly control the intensity of exercise, whether exercise occurs in a fasted or fed state, and measure gut peptides, food reward value, and food intake are needed to test the mechanistic model and demonstrate causality. Repeated, within day assessments are particularly needed to model dynamic changes in gut peptides. Across these designs, individual differences that predict susceptibility to reduced food intake after MVPA (King et al. 2017) are needed to inform treatment and predict risk. Consistent with the conceptualization of anorexia nervosa as a metabo‐psychiatric disorder (Bulik et al. 2019; Watson et al. 2019), it may be that increased susceptibility to the appetite‐suppressant effects of MVPA sets the stage for the development of anorexia nervosa and other restrictive eating disorders.

Clinical implications vary based on the population. For individuals engaging in severe dietary restriction, cardiovascular exercise intensity may need to be reduced, food consumption prior to MVPA may need to be prioritized, physical activity may need to be shifted from cardiovascular exercise to resistance training, and/or post‐MVPA caloric intake may need to be increased. Identifying multiple pathways has great clinical utility, particularly in instances where patients may be unwilling to give up MVPA and/or when exercise may be beneficial in addressing mood concerns. Understanding how exercise is linked to food intake may also have implications for the type of exercise to incorporate in treatments for binge‐eating disorder. Tailoring the types of exercise to presenting concerns or individual differences in susceptibility to MVPA‐induced dietary restriction may prove beneficial to improving treatment outcomes and facilitating healthy engagement in exercise.

## Author Contributions

**K. Jean Forney:** conceptualization, writing – original draft, writing – review and editing. **Angela R. Hillman:** conceptualization, writing – original draft, writing – review and editing. **Lindsay P. Bodell:** conceptualization, writing – original draft, writing – review and editing.

## Conflicts of Interest

The authors declare no conflicts of interest.

## Data Availability

Data sharing is not applicable to this article as no new data were created or analyzed in this study.
